# Familial multiple discoid fibromas: clinical features and a brief overview of the literature

**DOI:** 10.1016/j.abd.2025.501268

**Published:** 2026-01-07

**Authors:** Ece Gokyayla, Kamer Gunduz, Peyker Temiz

**Affiliations:** a Department of Dermatology and Venereology, Uşak Training and Research Hospital, Uşak, Turkey; b Department of Dermatology and Venereology, Manisa Celal Bayar University, Manisa, Turkey; c Department of Pathology, Manisa Celal Bayar University, Manisa, Turkey

Dear Editor,

Familial Multiple Discoid Fibromas (FMDF) is an extremely rare genodermatosis (OMIM 190340) presenting clinically with multiple, skin-coloured, dome-shaped papules located mainly on the face, neck, trunk and especially on pinna.^1^ Although FMDF resembles Birt-Hogg-Dubé (BHD) syndrome (OMIM 135150) clinically, it has been recognized as a distinct entity based on its specific clinical characteristics and genetic profile.^1^, ^2^

The index patient is a 25-year-old man who presented with multiple asymptomatic papules on his face, ears, trunk, and extremities. The lesions had begun in early adolescence and increased in number and size since then. Dermatological examination revealed multiple, firm, skin-coloured small papules on his central face, most prominently on the pinna (Fig. 1), trunk, and lower extremities. Histopathological examination revealed well-circumscribed discoid fibrovascular proliferation compatible with discoid fibroma (Fig. 2). No mutations were detected in genetic analysis of FLCN, PTEN, TSC1 and TSC2 loci. Radiological imaging of lungs, kidneys and brain was normal. When the family of the patient was evaluated, there was no parental consanguinity; the patient had only one brother. Similar multiple papules on the central face and the pinnae (Fig. 3) of his mother (47-year-old), and fewer papules on the face, pinnae and the neck of his younger brother (22-year-old) were detected. Their lesions had also begun in early adolescence. Histopathologic, genetic, and radiologic results of his mother and brother were similar.

**Figure 1:**
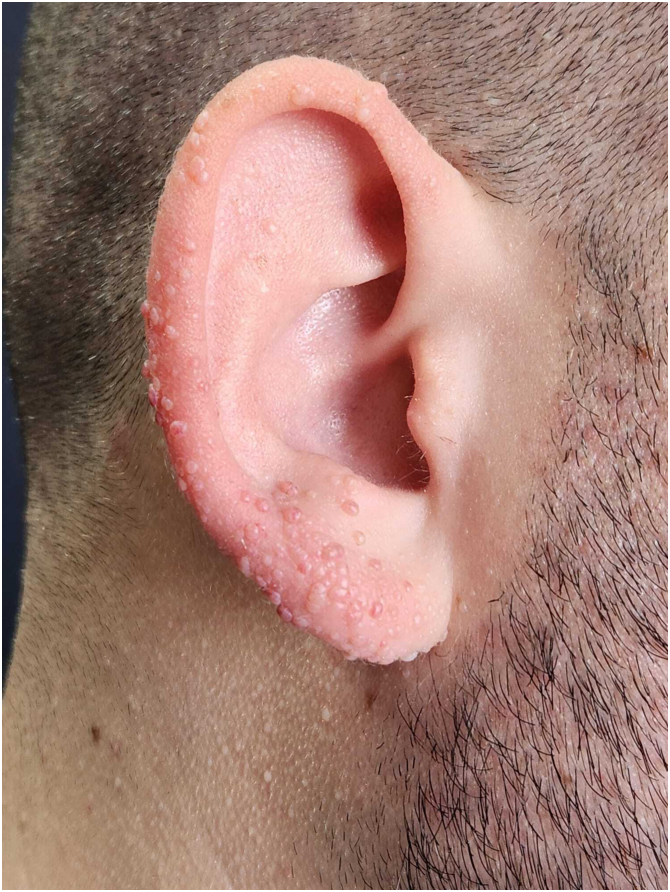
Discoid fibromas in Patient nº 1. Multiple skin-coloured small papules on the pinna.

**Figure 2:**
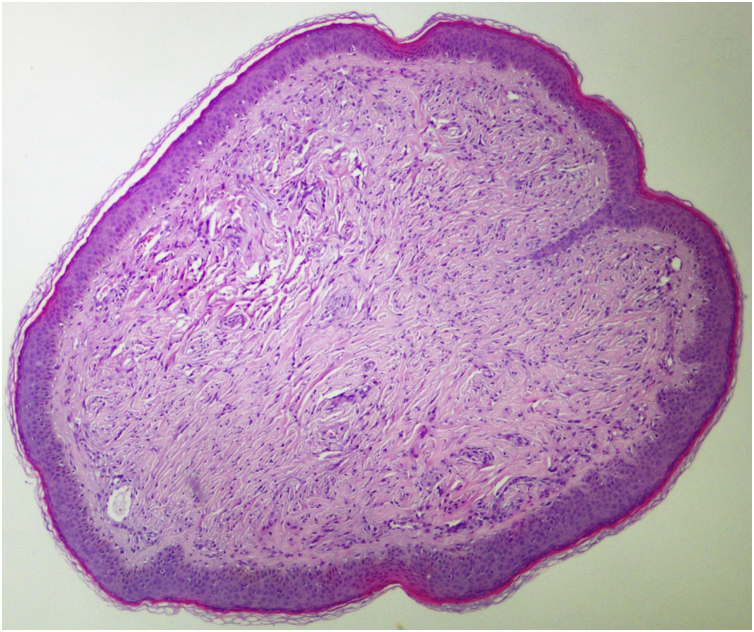
Histopathological examination of discoid fibroma. Well-circumscribed discoid fibrovascular proliferation (Hematoxylin & eosin, ×40).

**Figure 3:**
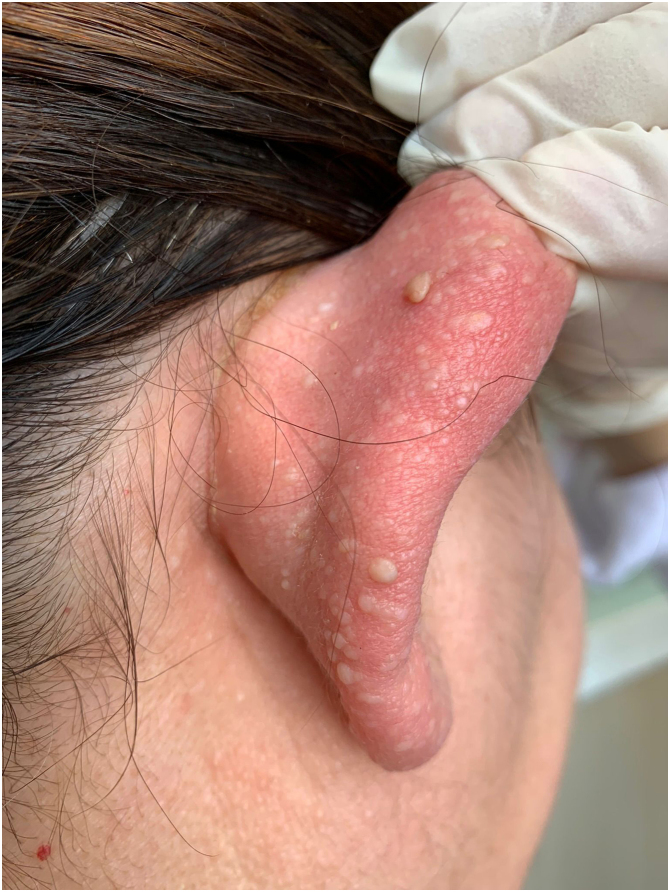
Discoid fibromas in his mother. Multiple skin-coloured papules on the pinna.

The literature review and patient characteristics are summarized in Table 1. Clinical differential diagnoses of multiple skin-coloured papules include BHD syndrome, multiple fibrofolliculomas, trichoepitheliomas, tuberous sclerosis complex, Cowden syndrome, and Brooke-Spiegler syndrome.^1^, ^2^, ^3^, ^4^ FMDF differs from these conditions by its characteristic early age of onset, preferential involvement of the pinna, distinct histopathology compatible with discoid fibroma, absence of systemic manifestations, and its different genetic profile.

**Table 1:** Overview of familial multiple discoid fibroma cases in literature.

Reference	Nº of Families and Patients	Age at Onset	Lesion Distribution and Morphology	Histopathology	Genetic Findings	Systemic Findings	Management and Outcomes
Starink et al. (J Am Acad Dermatol, 2012)	9 families (27 patients)	Childhood or adolescence (some as early as 1–3 years)	Mostly on the ear pinnae and central face (nose, cheeks). Lesions are 1–4 mm, dome-shaped fibrous papules, often with telangiectasia.	Well-circumscribed fibrovascular proliferations (“discoid fibromas”). No follicular epithelial proliferation as seen in fibrofolliculoma. Often a bent hair follicle at the periphery.	No pathogenic FLCN mutations. Linkage to BHD locus excluded by segregation analysis.	Rare sporadic renal cysts but no renal tumors. No pneumothorax.	Primarily surgical or destructive methods (eg, excision, curettage) for cosmetic reasons. Benign course without organ involvement. Emphasizes clinical/genetic distinction from BHD.
Wee et al. (Br J Dermatol, 2013)	2 siblings (no other affected relatives)	Brother at age 5; sister in adolescence.	Face (nose, malar area) and ear pinnae. Fewer lesions on trunk and limbs. Multiple firm papules.	“Discoid fibroma” architecture. Some lesions exhibit a unique “keloidal-like” pattern: thick hyalinized collagen bands surrounded by spindle or histiocyte-like cells. No fibrofolliculoma-like epithelial strands.	FLCN negative. TSC1/TSC2 negative.	No pulmonary or renal lesions. No evident familial pattern (possible autosomal recessive or germline mosaic).	Surgical approaches yielded suboptimal cosmetic outcomes. Topical rapamycin (1 mg/mL) once daily led to marked papule regression.
Tong et al. (Cureus, 2017)	15 families (44 patients), 1 de novo 39-year-old patient	Typically childhood or early adulthood (variable among families)	Mostly on the face (cheeks, nose) and ear pinnae, sometimes trunk. Small firm papules, occasionally telangiectatic.	Benign fibrous lesions, described as “trichodiscomas” or “discoid fibromas”. No central epithelial strands typical of fibrofolliculoma; peripheral hair follicle may be present.	FLCN mutation usually negative or benign variants. No pathogenic folliculin changes found.	Simple renal cyst in one case. No pneumothorax or renal tumors. No systemic malignancy.	Benign lesions; excisional or ablative treatments mainly for cosmetic reasons. No malignant transformation reported.
van de Beek et al. (J Hum Genet, 2023)	10 families	Not specifically stated.	Face, ear pinnae. Multiple “trichodiscomas” clinically described as FMDF.	Reported as consistent with “trichodiscoma/discoid fibroma” spectrum. No fibrofolliculoma-type epithelial strands.	FLCN negative. Linkage analysis reveals a locus on chromosome 5 (including FNIP1) in 9/10 families. A FNIP1 frameshift variant detected in most families; also a rare missense variant in PDGFRB co-segregating.	No mention of organ involvement.	No specific management details.

BHD, Birt-Hogg-Dubé syndrome; FLCN, Folliculin Gene; FMDF, Familial Multiple Discoid Fibroma; FNIP1, Folliculin Interacting Protein 1; mTOR, mammalian Target of Rapamycin; PDGFRB, Platelet-Derived Growth Factor Receptor Beta; TSC, Tuberous Sclerosis Complex.

Genetically, FMDF is defined by the absence of FLCN mutations, though a recent study suggests a possible link with a locus on chromosome 5 including the FNIP1 gene.^4^ Although it is not exactly settled, autosomal-dominant inheritance has been described for most of the patients. Also, in the family we reported, the fact that the mother and both of her male children were affected, despite no affected individuals in the ancestors, suggests that a de novo mutation in the mother was inherited in an autosomal dominant manner. The observation that the number and size of discoid fibromas were highest in the oldest patient ‒ the mother ‒ and lowest in the youngest patient ‒ the brother ‒ suggests that the disease burden accumulates progressively over time.

Regarding treatment, surgical destructive methods may be used in the treatment of discoid fibromas. Topical rapamycin was reported to be beneficial in a case report.^3^ We performed electrosurgery for the lesions on the pinnae in our patients. However, during follow-up, the lesions showed a tendency to recur.

In conclusion, our findings emphasize that clinical recognition and careful genetic characterization remain key challenges in FMDF. A better understanding of its genetic background could improve diagnosis, counseling, and therapeutic strategies for this rare entity.

## ORCID ID

Kamer Gunduz: 0000-0002-1319-9237

Peyker Temiz: 0000-0001-6308-0157

## Research data availability

Does not apply.

## Financial support

None declared.

## Authors' contributions

Ece Gokyayla: Contributed to the study conception and planning; effective participation in research orientation; critical literature review; data analysis and interpretation, and preparation and writing of the manuscript. She also performed a manuscript critical review and gave approval of the final version of the manuscript.

Kamer Gunduz: Contributed to the critical literature review; data collection, analysis and interpretation; preparation and writing of the manuscript, and performed manuscript critical review. She also gave approval of the final version of the manuscript.

Peyker Temiz: Contributed to data collection, analysis and interpretation and gave approval of the final version of the manuscript.

## Conflicts of interest

None declared.
